# Wideband MIMO Antenna System Employing Slot and Via Loading Technique for 5G Terminals

**DOI:** 10.3390/s26092745

**Published:** 2026-04-29

**Authors:** Xin-Hao Ding, Liang-Jun Zhan, Zhen Tan, Shah Nawaz Burokur

**Affiliations:** 1 School of Information Science and Technology, Nantong University, Nantong 226019, China; xhding@ntu.edu.cn (X.-H.D.);; 2 LEME, Univ Paris Nanterre, 92410 Ville d’Avray, France

**Keywords:** 5G, mobile terminal, multiple-input multiple-output (MIMO), wideband

## Abstract

**Highlights:**

**What are the main findings?**

- A four-element multiple-input multiple-output (MIMO) antenna system achieves wideband operation spanning from 4.23 GHz to 5.96 GHz. This tri-mode wideband operation is realized by independently controlling the TM_10_, TM_01_, and TM_11δ_ modes through the specific utilization of a slot and a pair of metallized vias within the patch element.
- The strategic implementation of an additional group of metallized vias effectively controls mutual coupling between the elements. This decoupling technique secures a high inter-element isolation level of over 17 dB, alongside a total efficiency exceeding 45% and an envelope correlation coefficient (ECC) lower than 0.25.

**What are the implications of the main findings?**

- The proposed multimode excitation technique provides sufficient bandwidth to practically cover multiple commercial frequency bands simultaneously, including N79 (4.4–5 GHz), V2X (5.905–5.925 GHz), and Wi-Fi 5/6 (5.150–5.850 GHz).
- By resolving the dependency on air spacing found in previous wideband multimode designs, this compact geometric configuration offers a highly efficient and practical solution that can be seamlessly and directly integrated onto the back covers of 5G mobile terminals.

**Abstract:**

This work introduces a wideband four-element multiple-input multiple-output (MIMO) antenna system with four rectangular patches arranged in a sequentially rotated configuration. Wideband frequency operation is realized by exploiting the TM_10_, TM_01_ and TM_11δ_ modes through the utilization of a slot and metallized vias in the patch design. Another group of metallized vias are used to control coupling between the antenna elements, achieving an isolation level of over 17 dB. A prototype is fabricated and measured, demonstrating −6 dB impedance bandwidth ranging from 4.23 GHz to 5.96 GHz, enabling coverage of the N79 (4.4–5 GHz), V2X (5.905–5.925 GHz) and Wi-Fi 5/6 (5.150–5.850 GHz) frequency bands. The MIMO antenna features an efficiency of over 45% and a low envelope correlation coefficient (ECC) lower than 0.25. Owing to its broad bandwidth, compact geometry, and good isolation, the proposed MIMO antenna provides an efficient and practical solution for 5G MIMO applications integrated within mobile terminal back covers.

## 1. Introduction

With the rapid development of fifth-generation (5G) communication systems, the demand for expanded spectrum resources, higher data rates, and enhanced data-handling capabilities in mobile terminals has become increasingly urgent. One of the most effective strategies to improve wireless channel capacity is to incorporate a larger number of antenna elements into the terminal architecture [1]. Consequently, multiple-input multiple-output (MIMO) technology has attracted extensive research attention, as it leverages spatial multiplexing to significantly increase data throughput without requiring additional spectral resources or higher transmission power [2,3,4]. Moreover, MIMO can improve link reliability and reduce fading effects by exploiting spatial diversity, which is particularly important in dense urban environments and high-mobility scenarios. In mobile terminals, MIMO arrays can be integrated either along the device frame or directly onto the back cover. A wide variety of frame-mounted MIMO implementations have been reported, including monopole, patch and slot-based designs [5,6,7,8,9,10]. While frame-mounted solutions offer certain mechanical and design conveniences, the continuous increase in the number of antennas—and consequently, the demand for higher-order MIMO configurations—has led to significant space limitations along the device frame. This spatial constraint can adversely affect element spacing, mutual coupling, and overall antenna performance. As a result, the utilization of the back cover as an alternative platform for antenna integration has gained considerable attention in recent years. Back-cover integration not only alleviates the space constraint on the frame but also provides a planar surface suitable for arranging multiple elements with sufficient separation, enabling better isolation, wider bandwidth, and improved MIMO performance. Additionally, the back cover offers opportunities for seamless integration with the device’s aesthetic and structural design, making it a practical and increasingly popular solution for modern 5G mobile terminals.

Meanwhile, efforts have been devoted to achieve desired frequency operations. As such, multiple resonant modes have been exploited to achieve operation within the N79 (4.4–5 GHz) frequency band [11] and multimode characteristics have been used to cover several commercial bands [12,13]. However, sufficient port isolation necessitates relatively large inter-element spacing, which consequently reduces the efficiency of space utilization. Thus, several MIMO antenna designs with enhanced isolation have been proposed [14,15,16,17]. In [18], a pair of degenerate characteristic modes is employed in combination with a capacitively coupled feeding network to achieve an isolation level above 20 dB, while in [19], a bent slot structure is utilized to enhance port isolation by leveraging the cancellation between electric and magnetic coupling paths. In [20], metallized vias positioned on the central radiating patch have been implemented to ensure an isolation exceeding 12.5 dB. Although the isolation has been effectively enhanced, the bandwidth remains limited for mobile terminal applications. To solve this issue, four PIFAs have been positioned in a sequentially rotated configuration to cover the N77 (3.3–4.2 GHz) frequency band [21]. In [22], a trapezoidal PIFA, a shorted trapezoidal monopole, and an L-shaped monopole branch have been utilized to provide three operational modes to cover the N77/N78/N79 bands. In [23], three resonant modes (the monopole mode, TM_10_, and TM_01_) are simultaneously excited to achieve wide bandwidth. Also, a MIMO configuration that effectively excites seven resonant modes across a wide operating range of 5.14–9.05 GHz has been presented [24]. However, two major limitations remain: first, in these multimode designs, the inability to independently control operational modes increases the design complexity, and second, the designs incorporate air spacing, which hinders direct integration onto the back cover of mobile terminals. In contrast, the proposed design uses a single-layer epoxy FR4 substrate without any air layer, achieving wideband operation covering N79, V2X, and Wi-Fi 5/6 simultaneously, constituting a distinct advantage over most existing wideband back-cover antennas that rely on an air gap [21,22,23,24], and offering a truly integrable solution for 5G mobile terminals.

This paper presents a compact four-element MIMO antenna designed for seamless integration onto the back cover of mobile terminals. Each patch element supports three resonant modes—TM_10_, TM_01_, and TM_11δ_—which are selectively controlled through a combination of a slot and a pair of metallized vias, enabling tri-mode wideband operation that covers key sub-6 GHz communication bands such as N79, V2X, and Wi-Fi 5/6. The slot facilitates mode separation and fine-tuning of resonant frequencies, while the vias provide inductive loading to optimize bandwidth and radiation performance. To further improve MIMO performance, an additional pair of vias is strategically introduced to suppress mutual coupling between adjacent elements, resulting in inter-element isolation exceeding 17 dB, low envelope correlation coefficients, and robust spatial diversity. The proposed antenna demonstrates a compact footprint, wide operational bandwidth, and high isolation, offering a practical and scalable solution for high-performance back-cover integration in next-generation 5G mobile terminals.

Compared with three-dimensional or frame-mounted antenna structures, planar back-cover designs such as the one proposed in this study offer significant advantages in terms of fabrication simplicity, mechanical integration, and low profile. For instance, though the 3D Vivaldi base-station array presented in [25] achieves high gain and dual polarization it is not suitable for direct integration into mobile terminal back covers due to its considerable thickness and assembly complexity. Our work focuses on a fully planar, low-cost and scalable solution, which are features that meet the stringent space constraints of modern smartphones and other communicating mobile devices.

## 2. Antenna Design

**Figure 1:**
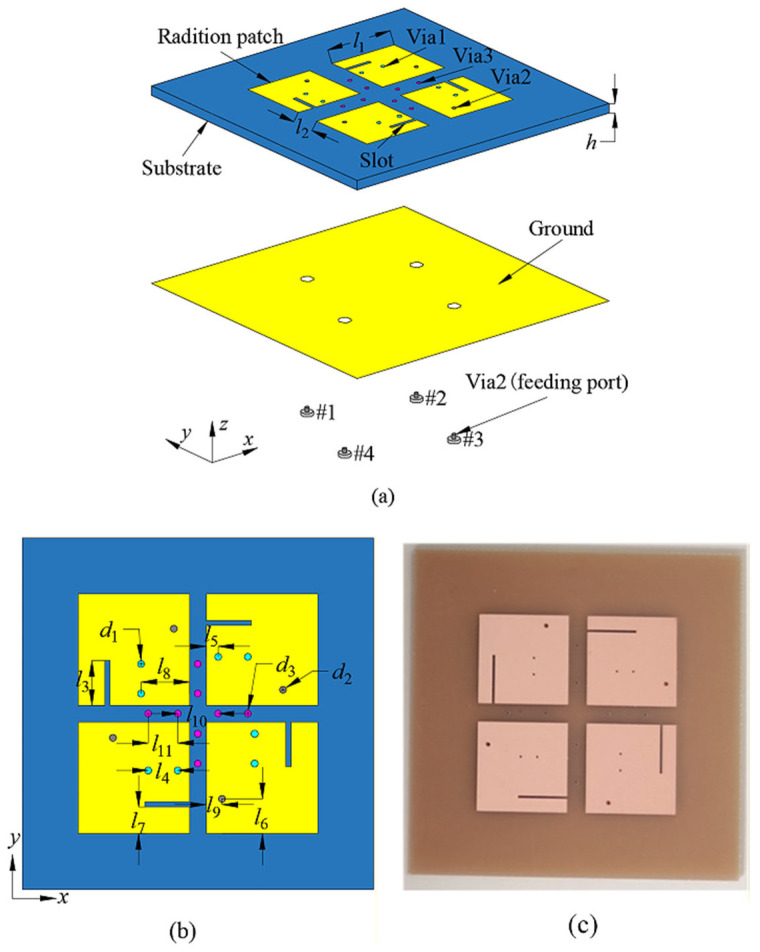
Structure of the proposed antenna: (**a**) 3D schematic design of the antenna, (**b**) top view of the antenna, and (**c**) photograph of the fabricated prototype. The design parameters are (units are in mm): *l*_1_ = 14.3, *l*_2_ = 3, *l*_3_ = 7.8, *l*_4_ = 2.7, *l*_5_ = 4.8, *l*_6_ = 1.7, *l*_7_ = 2.2, *l*_8_ = 7.3, *l*_9_ = 3.6, *l*_10_ = 10, *l*_11_ = 6, *d*_1_ = 0.2, *d*_2_ = 0.4, *d*_3_ = 0.2, and *h* = 2.

As depicted in Figure 1, the proposed design realizes a compact four-element MIMO antenna system suitable for integration in mobile terminals. The antenna is constructed on a standard epoxy FR4 dielectric substrate, featuring a relative permittivity of *ε*_r_ = 4.4 and a loss tangent of tan *δ* = 0.02, providing a cost-effective and mechanically stable platform. The overall architecture integrates four microstrip-etched radiating patches on the top surface, each carefully engineered to support multiple resonant modes, along with three sets of metallized vias: four pairs of Via1, four feeding vias (Via2), and four pairs of Via3 embedded within the substrate. Each patch includes a slot and a corresponding pair of Via1, where the slot, together with Via1, enables precise tuning of the fundamental TM_10_ mode, while the higher-order TM_11δ_ mode is predominantly controlled by Via1. This arrangement allows independent excitation of multiple modes, achieving wideband operation within a compact footprint. The fully metallized ground plane on the bottom of the substrate ensures proper current return paths and radiation efficiency. To further improve MIMO performance, a pair of additional metallized vias (Via3) is strategically inserted between adjacent patches to suppress mutual coupling and enhance inter-element isolation. Each patch is individually fed through a dedicated connector (Via2), ensuring uniform excitation and robust multi-port operation. Collectively, this design combines compact size, wideband tri-mode performance, and high isolation, making it a practical solution for high-performance MIMO deployment in modern mobile terminals.

### 2.1. Elementary Antenna Design

**Figure 2:**
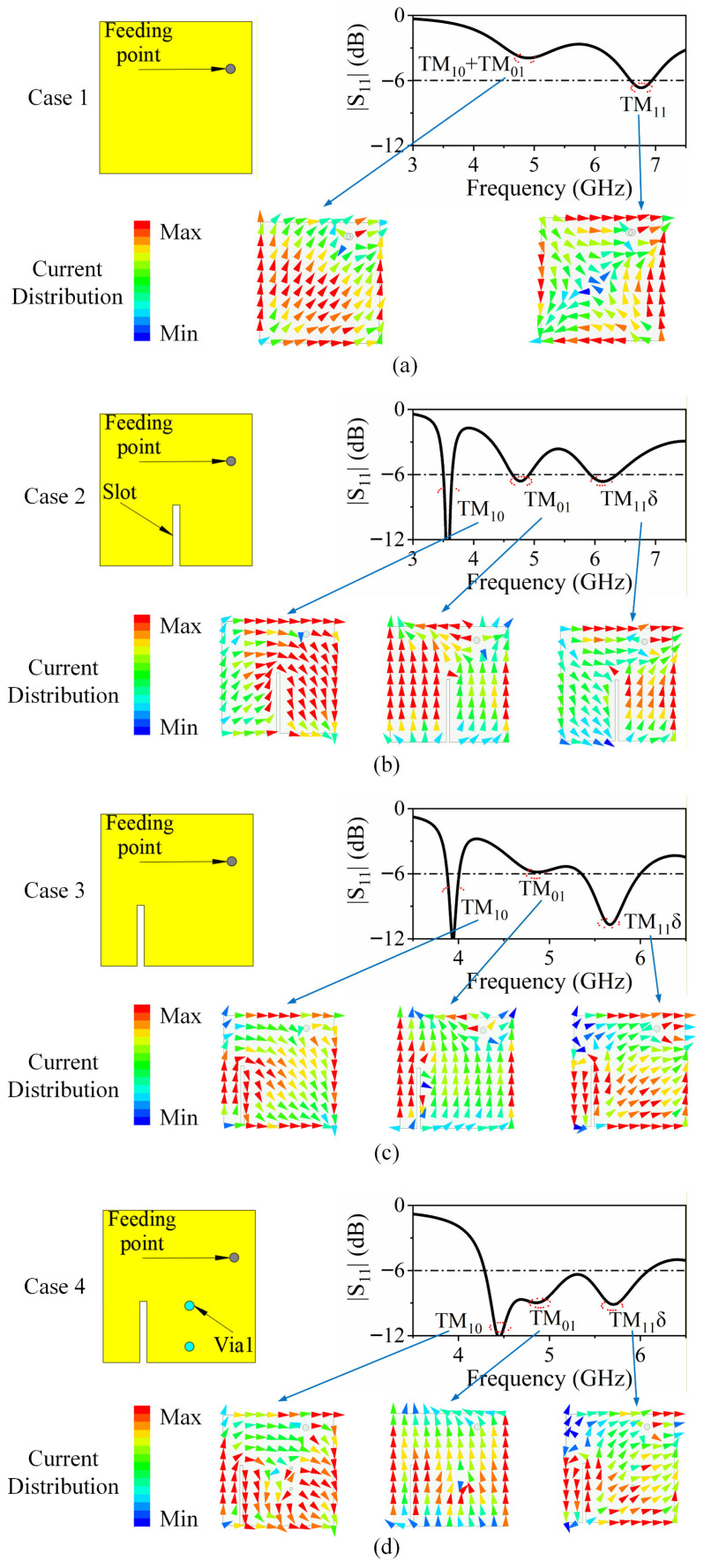
Evolutionary design process of the patch element along with the simulated bandwidth and current distribution characteristics of the operation mode: (**a**) Case 1, (**b**) Case 2, (**c**) Case 3, and (**d**) Case 4.

Figure 2 illustrates the evolutionary design process of the elementary patch element, along with the corresponding simulated bandwidth and surface current distribution characteristics for each design stage (Cases 1 to 4). The simulation process is conducted using ANSYS HFSS (2023 R2) [26] with a driven modal solution. Radiation boundaries are placed at least *λ*/4 away from the structure, with *λ* corresponding to the lowest frequency of interest, and lumped 50 Ω ports are placed at the feeding vias. In Figure 2a, Case 1 presents the initial patch configuration, which supports a hybrid TM_10_/TM_01_ mode alongside the TM_11_ mode. Analysis of the current distribution reveals that the hybrid TM_10_/TM_01_ mode and the TM_11_ mode resonate at 4.8 GHz and 6.8 GHz, respectively, indicating partial mode degeneracy in the fundamental modes. To achieve independent control of the TM_10_ and TM_01_ modes, a narrow slot is introduced on the patch in Case 2, as shown in Figure 2b. This slot effectively elongates the current path associated with the TM_10_ mode while leaving the TM_01_ mode largely unaffected. As a result, the previously degenerate hybrid modes are separated, with the TM_10_ mode resonating at 3.6 GHz and the TM_01_ mode remaining near 4.8 GHz. The introduction of the slot also influences the higher-order TM_11_ mode, causing a downward shift in its resonance to 6.1 GHz. Additionally, the current distribution of this mode exhibits noticeable distortion due to the modified patch geometry, prompting its reclassification as the TM_11δ_ mode, where δ indicates a distorted or shifted mode relative to the pure TM_11_.

However, the TM_10_ and TM_11δ_ modes remain inherently coupled, making their independent control challenging and limiting the realization of multimode wideband operation. To address this issue, Cases 3 and 4 introduce structural modifications aimed at decoupling these two modes. In Figure 2c, Case 3 demonstrates that shifting the slot away from the patch center results in a higher resonant frequency for the TM_10_ mode, while simultaneously lowering the resonant frequency of the TM_11δ_ mode. This behavior arises because the slot offset creates a region with reduced TM_10_ current intensity and an area where TM_11δ_ currents are more concentrated. Consequently, compared to Case 2, the offset slot reduces its effect on the TM_10_ mode while enhancing its influence on the TM_11δ_ mode, thereby enabling partial independent tuning of these coupled modes.

**Figure 3:**
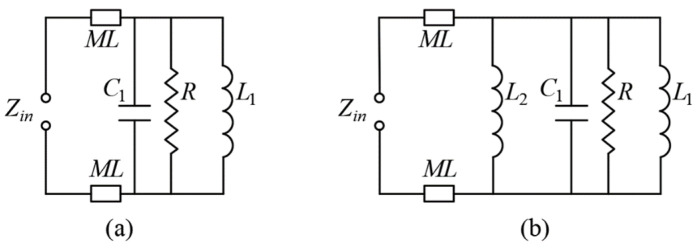
Equivalent *RLC* resonant circuit of TM_10_ in (**a**) Case 3 and (**b**) Case 4.

Subsequently, as illustrated in Figure 2d, for Case 4, two metallized vias (Via1) are strategically incorporated along the *y*-axis of the antenna structure. Due to their orientation, these vias primarily interact with the *x*-polarized mode, specifically influencing the TM_10_ resonant mode of the patch. Functionally, the vias introduce additional inductance to the TM_10_ mode, effectively modifying its resonant behavior. To analyze this effect quantitatively, the TM_10_ mode without and with Via1 can be equivalently modeled as two parallel *RLC* resonant circuits, as depicted in Figure 3. The resonant frequency of these equivalent circuits can be expressed using the standard *RLC* parallel resonance formula. For the TM_10_ mode without Via1, the resonant frequency *f_a_* is determined by the inherent resistance *R*, inductance *L*_1_, and capacitance *C*_1_ of the patch, representing the natural electromagnetic response of the structure. When Via1 is introduced, it provides an additional inductance *L*_2_ in parallel with *L*_1_. The combined parallel inductance reduces the overall inductive reactance of the TM_10_ branch, thereby increasing the resonant frequency from *f_a_* to *f_b_*. This effect can be viewed as a frequency tuning mechanism, allowing precise control of the TM_10_ resonance by adjusting the physical dimensions, positions, and number of vias. Moreover, the inclusion of Via1 minimally perturbs the other resonant modes (TM_01_ and TM_11δ_), demonstrating selective mode control, which is particularly advantageous for multi-mode wideband antenna design. The *RLC* equivalent circuit analysis provides an intuitive and accurate framework to predict the shift in resonant frequency due to structural modifications, bridging the physical design and theoretical modeling.

(1) $$f_{a}=\frac{1}{2\pi \sqrt{L_{1}C_{1}}}$$

(2) $$f_{b}=\frac{1}{2\pi \sqrt{\frac{L_{1}L_{2}}{L_{1}+L_{2}}C_{1}}}$$

The addition of Via1 effectively shifts the TM_10_ mode toward a higher frequency, closer to the TM_01_ mode, while its influence on the TM_11δ_ mode is negligible. This selective tuning allows for precise control over the TM_10_ mode without disturbing the higher-order TM_11δ_ mode. As a result, the three modes—TM_10_, TM_01_, and TM_11δ_—can be simultaneously excited and independently controlled, achieving a tri-mode wideband operation with reflection coefficient *S*_11_ < –6 dB over a frequency range from 4.28 GHz to 6.08 GHz. This frequency coverage encompasses the N79 band (4.4–5 GHz), V2X band (5.905–5.925 GHz), and Wi-Fi 5/6 channels (5.15–5.85 GHz), making the design suitable for multi-standard terminal applications.

**Figure 4:**
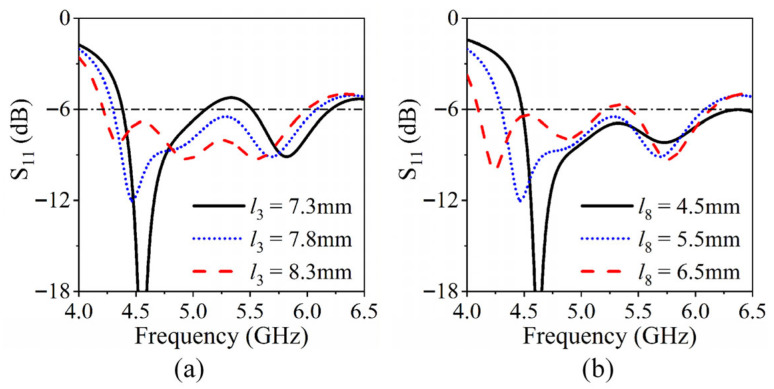
Influence of (**a**) the slot length *l*_3_, and (**b**) the position of Via1 (*l*_8_) on *S*_11_ response of the antenna.

A further parametric study is conducted to investigate the effects of the slot length *l*_3_ and the position *l*_8_ of Via1 on the *S*_11_ response, as illustrated in Figure 4. Increasing *l*_3_ causes both the TM_10_ and TM_11δ_ modes to shift toward lower frequencies, reflecting the elongation of the effective current path and enhanced coupling to the higher-order mode. In contrast, increasing *l*_8_ selectively downshifts only the TM_10_ mode, demonstrating the ability of Via1 to independently regulate the fundamental mode without impacting the TM_11δ_ resonance.

### 2.2. MIMO Antenna Design

**Figure 5:**
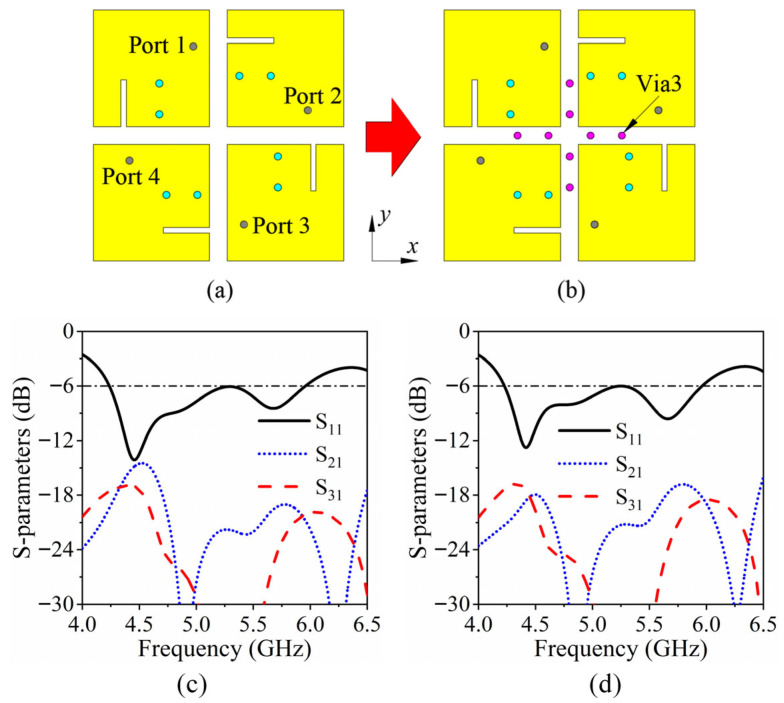
Decoupling design of the MIMO system: (**a**) Case 4, (**b**) Case 5. Simulated *S*-parameters of the MIMO system: (**c**) Without Via3, and (**d**) With Via3.

As presented in Figure 5a, the four-element MIMO antenna is meticulously designed by arranging the four patches in a sequentially rotated configuration, where each individual patch is independently excited through its corresponding feeding port. This rotational arrangement not only ensures spatial diversity but also enhances the orthogonality of the radiated fields, which is critical for mitigating mutual coupling and improving MIMO system performance. Owing to the structural symmetry of the antenna, the electromagnetic behavior and performance characteristics of the entire array can be effectively represented by analyzing Element 1, significantly reducing computational complexity while maintaining accuracy in predicting array performance.

As shown in Figure 5c, the isolation between Port 1 and Port 3 exceeds 17 dB, which can be attributed primarily to the relatively larger physical separation between these two elements, as well as the rotational arrangement that minimizes direct coupling paths. The isolation level indicates a sufficiently low mutual coupling for practical MIMO applications, ensuring minimal signal leakage and preserving the integrity of independent channels. In contrast, the isolation between Port 1 and Port 2 is approximately 14 dB, which is slightly lower due to the shorter inter-element distance. Despite this, the observed isolation remains within acceptable limits for multi-port MIMO operation, allowing for effective decoupling and low correlation between channels. Additionally, the current distribution analysis shows that the coupling between closely spaced elements is dominated by near-field interactions, whereas the coupling between diagonally positioned elements is mainly influenced by far-field radiation overlap, further explaining the difference in isolation levels. This detailed evaluation confirms that the proposed sequentially rotated configuration achieves a balanced trade-off between compact array size, spatial diversity, and acceptable isolation, providing a robust foundation for wideband MIMO performance in practical millimeter-wave terminal applications.

To further enhance port isolation and reduce the adverse effects of mutual coupling, Via3 structures are strategically incorporated between the antenna elements. These vias act as vertical conductive paths, providing an effective return route for induced currents and thereby suppressing surface-wave and near-field coupling between adjacent patches. In Case 5, as depicted in Figure 5b, four pairs of additional vias are introduced symmetrically, ensuring that the mitigation effect is uniform across all elements. The placement of these vias is carefully optimized to maximize decoupling without significantly perturbing the radiation pattern or input impedance of each antenna element. As illustrated in Figure 5d, the incorporation of the via structures results in a substantial improvement in isolation: the isolation between Port 1 and Port 2, as well as between Port 1 and Port 3, is increased to exceed 17 dB. This enhancement can be attributed to the suppression of both near-field coupling, which is dominant between closely spaced elements, and surface-wave propagation along the substrate. Considering the geometrical symmetry of the four-element MIMO configuration, it can be inferred that all other port pairs exhibit similar improvements, achieving isolation levels of at least 17 dB across the designated operating frequency band. Moreover, the addition of the vias minimally affects the reflection coefficients of individual ports, maintaining good impedance matching while achieving effective decoupling. This demonstrates that the proposed via-assisted design not only provides robust mutual coupling suppression but also preserves the intrinsic radiation characteristics of each antenna element, thereby enhancing the overall performance of the MIMO antenna system in practical millimeter-wave terminal applications.

In comparison with other decoupling techniques such as neutralization lines or defected ground structures (DGS) [27], the proposed via-based method presents the advantage of being fully embedded within the substrate, preserving the ground plane integrity and not increasing the antenna footprint. Moreover, though DGS can effectively suppress surface waves, they tend to participate in increasing backward radiation. While neutralization lines can achieve high isolation, they often require additional meandering lines that may cause unwanted resonances [28]. As such, our exploited via-only approach offers a simple and effective solution for low-profile MIMO arrays, which can further be processed using standard low-cost printed circuit board (PCB) fabrication technique. Regarding scalability to higher-order MIMO systems, such as 8 or 16 elements-based MIMO systems, the sequentially rotated 2 × 2 block can serve as an elementary building block. Multiple elementary blocks can then be tiled on the back cover of a larger terminal, for instance, tablet or phablet, with additional decoupling vias placed between the blocks to maintain good isolation.

**Figure 6:**
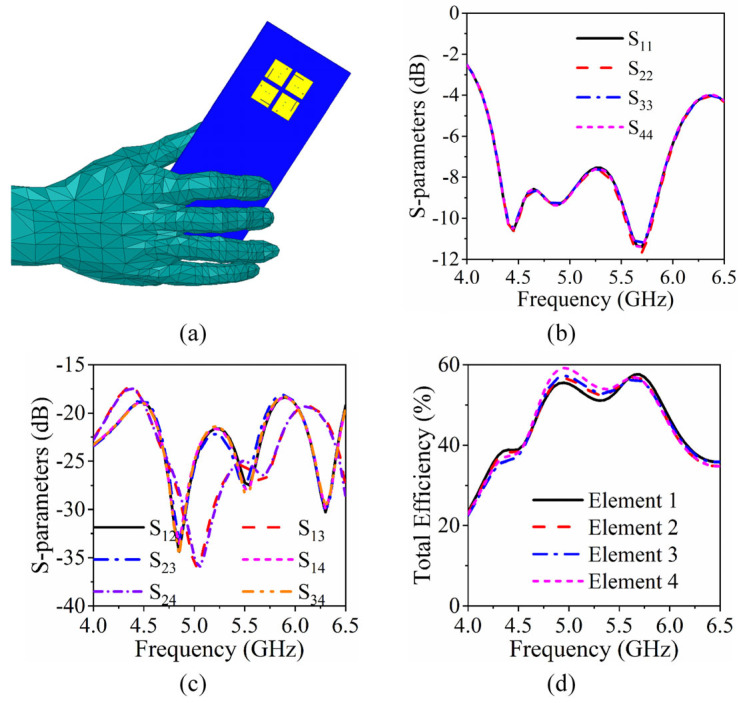
Influence of a user’s hand on the antenna performance. One-hand mode: (**a**) structure and corresponding simulated (**b**) bandwidths, (**c**) mutual couplings, and (**d**) total efficiency.

Specific absorption rate (SAR) referring to the electromagnetic power absorbed or consumed by the unit mass of biological tissue, is an influential parameter for assessing the radiation strength from a mobile terminal to the human body. The 10 g spatial average one-hand SAR of the proposed antenna is thus simulated. The evaluation model of a human hand holding a mobile phone is shown in Figure 6a. The simulated SAR peak values at 4.4 GHz, 4.8 GHz, and 5.8 GHz are found to be equal to 0.227, 0.733 and 0.38, respectively, which are below the SAR limit of 2.0 W/kg for 10-g human tissue, thus validating the compliance of the proposed antenna with safety standards and its suitability for applications in 5G mobile terminal devices.

To evaluate the antenna’s performances under practical user holding conditions, full-wave electromagnetic simulations are conducted in Ansys HFSS with a standard anatomical human hand model (compliant with IEEE Std 1528-2013 [29]), which was placed in a typical holding position with the palm in contact with the back of the smartphone and the fingers wrapped around the side edges. The influence of the human hand on the performances of the antenna is simulated, and the results are presented in Figure 6b,c. The −6 dB impedance bandwidth covers 4.25–6.0 GHz in both free-space and hand-holding scenarios. The isolation between antenna elements remains better than 17 dB across the entire operating band, which is almost unchanged compared with the free-space condition. Although the total efficiency is slightly deteriorated by the hand effect, it remains above 40% within the operating band, as illustrated in Figure 6d. These results confirm that the proposed antenna maintains good performance under practical user-holding conditions.

## 3. Experimental Verification

**Figure 7:**
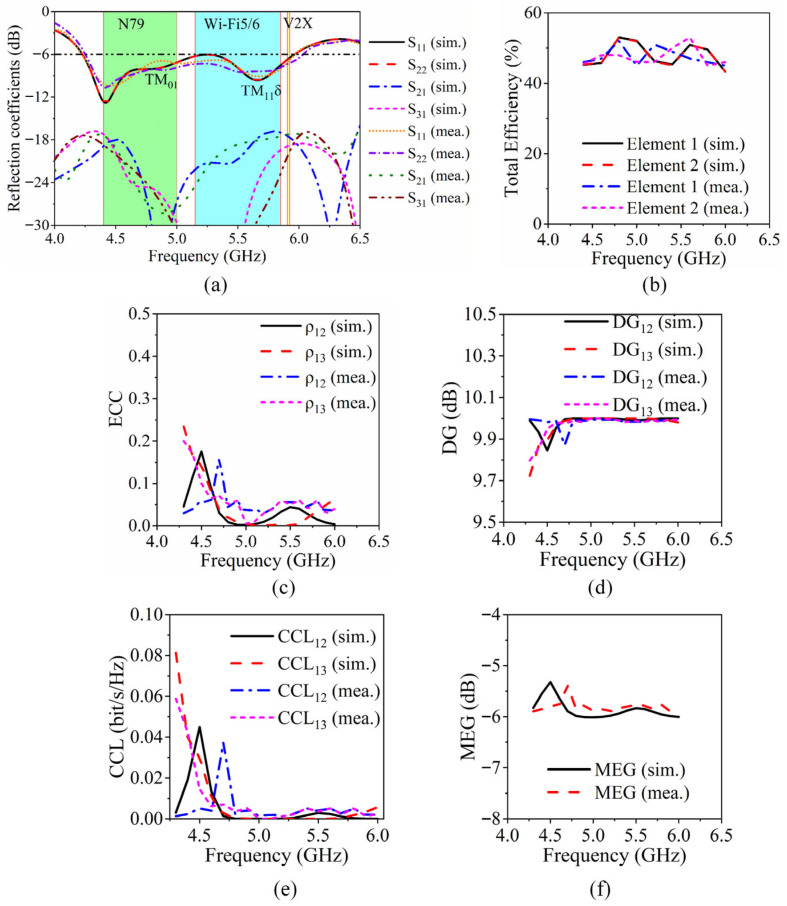
Performances of the proposed MIMO antenna system. Simulated and measured (**a**) *S*-parameters, (**b**) total efficiency, (**c**) ECC, (**d**) DG, (**e**) CCL, and (**f**) MEG.

The proposed four-element MIMO antenna is fabricated using standard PCB technique, as depicted in the photograph shown in Figure 1c, and subsequently subjected to comprehensive experimental validation. For the experimental validation of the performances, *S*-parameters are measured using an Agilent N5230C four-port differential vector network analyzer (VNA). The four antenna feeding ports are directly connected, using radiofrequency coaxial cables, to the four VNA ports after a full 4-port Short-Open-Load-Through (SOLT) calibration. Radiation patterns and total efficiency are measured in a microwave/millimeter-wave anechoic chamber (0.8 GHz–40 GHz range) using a 2–18 GHz standard horn antenna, as receiver. The simulated and measured *S*-parameters are presented in Figure 7a over the 4–6.5 GHz frequency band, clearly showing how the three resonant modes merge to cover N79, V2X, and Wi-Fi 5/6 simultaneously. Furthermore, a close agreement between simulation and experimental performances can be observed across the operating frequency bands. The measured −6 dB impedance bandwidth spans from 4.23 GHz to 5.96 GHz, corresponding to a fractional bandwidth of 34%, which aligns well with the simulated predictions. Moreover, the measured mutual coupling between antenna elements remains below −17 dB throughout the entire operational frequency range, confirming the effectiveness of the decoupling strategy and validating the design approach.

Figure 7b,c illustrates the total efficiencies and envelope correlation coefficients (ECC) of the MIMO antenna, respectively. The ECC can be calculated as follows [30]:

(3) $${\mathrm{ECC}}_{ij}=|\frac{\int \int_{4\pi }F_{i}(\theta ,\phi )\cdot F_{j}^{*}(\theta ,\phi )d\Omega }{\sqrt{\int \int_{4\pi }\left|\right|F_{i}|{|}^{2}d\Omega \cdot \int \int_{4\pi }\left|\right|F_{j}|{|}^{2}d\Omega }}{|}^{2}$$

$$\mathrm{with}\;F_{i}(\theta ,\phi )=E_{\theta ,i}(\theta ,\phi )\hat{\theta }+E_{\phi ,i}(\theta ,\phi )\hat{\phi }\;\mathrm{and}\;d\Omega =\sin\theta d\theta d\phi .$$

Equation (3) served as the basis to determine measured correlation values from the reflection coefficient measurements. The total radiation efficiency exceeds 45% for all ports, indicating minimal losses and effective power transfer, while the ECC values remain below 0.25 across the band, significantly lower than the widely accepted threshold of 0.5 [31]. These results confirm that the antenna elements operate with low correlation, supporting reliable spatial multiplexing and high MIMO diversity performance.

In addition to ECC and efficiency, other MIMO performance metrics have been evaluated, as presented in Figure 7d–f. When all four ports have the same phase, the diversity gain (DG) of the MIMO antenna can be calculated as follows [32]:

(4) $${\mathrm{DG}}_{ij}\approx 10\sqrt{1-|ECC_{ij}{|}^{2}}[\mathrm{dB}]$$

and the channel capacity loss (CCL) of the MIMO antenna can be calculated as follows [30]:

(5) $$\mathrm{CCL}=-\log_{2}(\det(\Psi )),\Psi_{ij}\;=\sum_{k=1}^{4}S_{ik}S_{jk}^{*}\;(i\neq j)$$

The DG of all antenna elements is close to the ideal value of 10 dB (ranging from 9.72 dB to 9.99 dB), and the CCL is far less than 0.1 bit/s/Hz (maximum CCL = 0.082 bit/s/Hz), suggesting that the proposed antenna has good diversity performance and high MIMO communication efficiency.

Moreover, the mean effective gain (MEG) is a significant metric to evaluate the MIMO diversity, since it highlights the performance of every antenna part separately in the multipath channel. MEG of the MIMO antenna is given as follows [33]:

(6) $${\mathrm{MEG}}_{i}\approx \frac{1}{4}(1-\sum_{j=1}^{4}\eta_{j}(1-ECC_{ij}))$$

The MEG values obtained from simulation and measurement are stably distributed in the ranges of −6 dB–−5.3 dB and −6 dB–−5.4 dB, respectively, and are found to be in good agreement.

**Figure 8:**
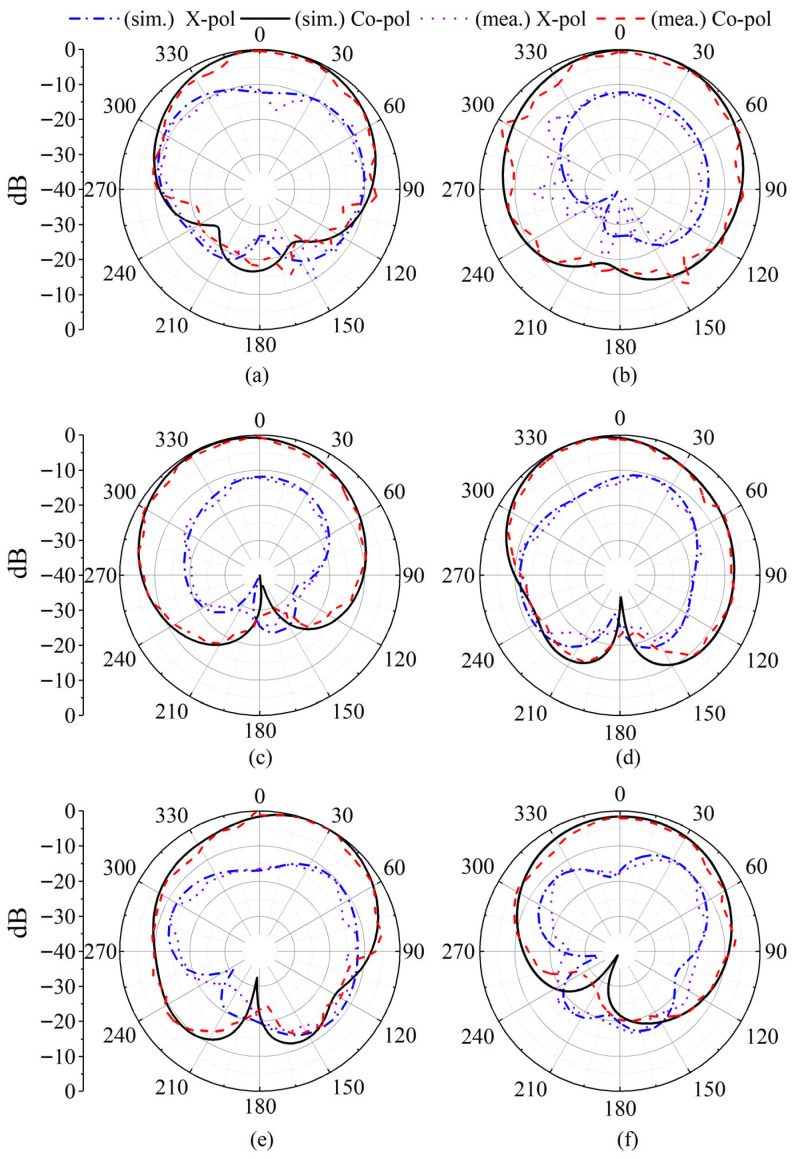
Simulated and measured radiation patterns of Element 1. (**a**) At 4.4 GHz in the *xoz* plane and (**b**) *yoz* plane. (**c**) At 4.8 GHz in the *xoz* plane and (**d**) *yoz* plane. (**e**) At 5.8 GHz in the *xoz* plane and (**f**) *yoz* plane.

Additionally, the measured and simulated far-field radiation patterns of Element 1 are presented at three representative frequencies 4.4 GHz, 4.8 GHz, and 5.8 GHz—in both the *xoz* and *yoz* planes, as shown in Figure 8. A strong agreement is observed between simulation and measurement results, verifying the accuracy of the electromagnetic model and the reliability of the fabrication process. A slight tilt of approximately 10° toward 330° is observed in the *yoz*-plane radiation pattern (Figure 8d), which is attributed to the asymmetrical placement of the slot and Via1 within the patch element. However, this slight angular tilt does not degrade the MIMO performance, as the envelope correlation remains low. Due to the inherent geometrical symmetry of the array, it can be inferred that the remaining antenna elements provide complementary radiation patterns, thereby ensuring robust polarization and spatial diversity. This structural symmetry, combined with the low mutual coupling and acceptable efficiency, guarantees comprehensive spatial coverage and reliable multi-channel performance, highlighting the suitability of the proposed design for practical MIMO terminal applications.

Table 1 provides a comprehensive performance comparison between the proposed four-element MIMO antenna and several representative designs reported in the literature. The antennas presented in [18,19,20] demonstrate relatively compact sizes, making them suitable for space-constrained applications. However, a closer inspection reveals certain limitations: the designs in [18,19] incorporate only two MIMO elements, which restricts their potential for higher-order spatial multiplexing, and the operational bandwidths reported in [18,19,20] are insufficient to cover multiple communication bands required in contemporary mobile terminals. On the other hand, the designs presented in [21,22,23,24] achieve wideband performance, yet the isolation between their MIMO elements is relatively limited, which may degrade the channel capacity and overall diversity performance in practical deployment. A critical observation is that, except for the design in [20], most of the existing antennas are not directly suitable for integration onto the back cover of a mobile terminal, due to constraints in form factor, thickness, or placement compatibility. In contrast, the proposed antenna demonstrates a holistic improvement across multiple key performance indicators. Specifically, it maintains a wide operational bandwidth sufficient to cover relevant sub-6 GHz communication bands, supports four MIMO elements to enhance spatial multiplexing capability, and achieves mutual coupling suppression such that the isolation exceeds 17 dB across the operating band. Meanwhile, it presents a low SAR value of less than 2 W/kg averaged over 10 g of human tissue. Moreover, its compact geometry and planar form factor allow for direct integration onto the back cover of a mobile device without additional spacing or structural modifications. This combination of wideband operation, high isolation, multi-element configuration, and seamless integration capability distinguishes the proposed design from prior work, making it particularly suitable for modern mobile terminals that demand high data rates, robust MIMO performance, and efficient use of limited space.

Finally, Table 2 studies the complexity of the structure by comparing the geometry, such as the number of vias, slots, substrate layers, and air layers, with those presented in previous studies. The main observation is that our proposed design uses a single epoxy FR4 layer without any air spacing layer and a moderate number of vias, for instance 4 pairs for mode control and 4 pairs for decoupling, which results in a simpler and ease of fabrication design than multilayer or air-gap involved designs.

## 4. Conclusions

In this paper, a compact wideband four-element MIMO antenna has been designed, fabricated, and experimentally validated. The proposed design leverages a combination of three resonant modes—TM_10_, TM_01_, and TM_11δ_—realized through the incorporation of carefully engineered slots and metallized vias, which collectively enable wideband operation. This multi-mode excitation strategy ensures coverage of key communication bands, including N79, V2X, and Wi-Fi 5/6, providing a versatile solution for contemporary mobile terminals. To further enhance the mutual coupling performance, additional metallized vias are strategically integrated between antenna elements, effectively suppressing surface-wave and near-field interactions, thereby achieving an isolation level exceeding 17 dB across the operating frequency band. The fabricated prototype demonstrates an impedance bandwidth spanning 4.23–5.96 GHz, with total radiation efficiency consistently above 45% for all ports and envelope correlation coefficient (ECC) values remaining below 0.25, confirming excellent MIMO performance and channel decorrelation. The antenna maintains a compact footprint, ensuring compatibility with the constrained form factors typical of modern mobile devices. In addition, the rotational symmetry and carefully optimized feeding network contribute to complementary radiation patterns across all four elements, enabling robust spatial diversity and high-quality coverage in practical deployment scenarios. Overall, the proposed MIMO antenna simultaneously achieves wideband operation, high isolation, compact size, and satisfactory efficiency, demonstrating its suitability for integration into 5G mobile terminals and other advanced wireless communication applications. The combination of multi-mode excitation, structural optimization, and decoupling techniques offers a practical and scalable approach for high-performance, multi-element antenna design in compact devices.

## Figures and Tables

**Table 1:** Performance comparison with the previous antenna designs.

Ref.	Bandwidth (GHz) for *S*_11_ = −6 dB		Total Efficiency (%)	Isolation (dB)	ECC	Electrical Dimensions ($\lambda_{L}^{3}$)	Number of Elements	10 g Peak SAR (W/kg)	Direct Integration Onto the Back Cover
[18]	3.28–3.85	15.9%	>52%	>24	<0.13	0.3 × 0.3 × 0.02	2	/	No
[19]	5.13–5.86	13.3%	>51%	>12	<0.21	0.25 × 0.25 × 0.034	2	/	No
[20]	4.26–5.12	18.3%	>48%	>12.5	<0.14	0.43 × 0.43 × 0.028	4	<1.96	Yes
[21]	3.3–4.2	24%	>36%	>9.7	<0.5	0.34 × 0.34 × 0.22	4	/	No
[22]	3.3–5	41%	>40%	>10	<0.13	0.46 × 0.46 × 0.22	4	<1.31	No
[23]	3.3–5	41%	>55%	>12	<0.05	0.65 × 0.65 × 0.028	4	/	No
[24]	5.14–9.05	55.1%	>44%	>12	<0.04	0.51 × 0.44 × 0.034	4	/	No
[30]	7.1–8.4	24.5%	64–82%	>17	<0.05	3.73 × 1.42 × 0.038	20	/	Yes
[34]	3.3–4.2	24%	>58%	/	/	0.22 × 0.35 × 0.017	2	/	Yes
[35]	6.42–7.12	10%	>54.7%	>15	<0.2	0.28 × 1.12 × 0.02	4	/	No
This work	4.23–5.96	34%	>45%	>17	<0.25	0.44 × 0.44 × 0.028	4	<0.74	Yes

*λ_L_* is the free-space wavelength at the lowest frequency.

**Table 2:** Complexity comparison among the proposed and prior works.

Ref.	Number of Vias	Number of Slots	Sub. Layers	Air Layers
[18]	48	28	2	1
[19]	14	19	2	1
[20]	28	4	1	0
[21]	24	8	2	1
[22]	16	8	2	1
[23]	40	8	2	1
[24]	52	32	2	1
[30]	0	120	1	0
This work	20	4	1	0

## Data Availability

The original contributions presented in the study are included in the article; further inquiries can be directed to the corresponding authors.

## References

[1] Ding X.-H., Cao Y., Yang W.-W., Chen J.-X. (2024). A compact microwave/millimeter-wave shared-aperture antenna with wideband millimeter-wave beam-steering ability. IEEE Antennas Wirel. Propag. Lett..

[2] Li M., Cheung S. (2021). Isolation enhancement for MIMO dielectric resonator antennas using dielectric superstrate. IEEE Trans. Antennas Propag..

[3] Fang Y., Jia Y., Zhu J.-Q., Liu Y., An J. (2024). Self-decoupling, shared-aperture, eight-antenna MIMO array with MIMO-SAR reduction. IEEE Trans. Antennas Propag..

[4] Qureshi U., Khan M.U., Sharawi M.S., Burokur S.N., Mittra R. (2021). Field decorrelation and isolation improvement in an MIMO antenna using an all-dielectric device based on transformation electromagnetics. Sensors.

[5] Sun L., Li Y., Zhang Z., Feng Z. (2020). Wideband 5G MIMO antenna with integrated orthogonal-mode dual-antenna pairs for metal-rimmed smartphones. IEEE Trans. Antennas Propag..

[6] Qu L., Kim H., Jung K.-Y., Liu Y. (2024). Compact four-port MIMO antenna module with decoupling components for 5G terminal devices. IEEE Antennas Wirel. Propag. Lett..

[7] Sun L., Li Y., Zhang Z. (2021). Wideband decoupling of integrated slot antenna pairs for 5G smartphones. IEEE Trans. Antennas Propag..

[8] Sun L., Li Y., Zhang Z. (2021). Wideband integrated quad-element MIMO antennas based on complementary antenna pairs for 5G smart-phones. IEEE Trans. Antennas Propag..

[9] Sun L., Li Y., Zhang Z., Wang H. (2020). Self-decoupled MIMO antenna pair with shared radiator for 5G smartphones. IEEE Trans. Antennas Propag..

[10] Yang B., Xu Y., Tong J., Zhang Y., Feng Y., Hu Y. (2022). Tri-port antenna with shared radiator and self-decoupling characteristic for 5G smartphone application. IEEE Trans. Antennas Propag..

[11] Cheng B., Du Z. (2022). A wideband low-profile microstrip MIMO antenna for 5G mobile phones. IEEE Trans. Antennas Propag..

[12] Chang L., Liu H. (2022). Low-profile and miniaturized dual-band microstrip patch antenna for 5G mobile terminals. IEEE Trans. Antennas Propag..

[13] Chang L., Zhang G., Wang H. (2022). Triple-band microstrip patch antenna and its four-antenna module based on half-mode patch for 5G 4×4 MIMO operation. IEEE Trans. Antennas Propag..

[14] Zhang A., Wei K., Hu Y., Guan Q. (2022). High-isolated coupling-grounded patch antenna pair with shared radiator for the application of 5G mobile terminals. IEEE Trans. Antennas Propag..

[15] Zhang A., Wei K., Chu S., Wang Y. (2022). Extremely compact interconnected half-mode cavity antennas with enhanced isolation for MIMO system. IEEE Trans. Antennas Propag..

[16] Zhang A., Wei K., Chu S., Guan Q., Hu Y. (2022). Low-profile patch antenna pair with pattern diversity using common and differential modes for MIMO systems. IEEE Antennas Wirel. Propag. Lett..

[17] Zhang A., Wei K., Zhang Z. (2023). Multiband and wideband self-multipath decoupled antenna pairs. IEEE Trans. Antennas Propag..

[18] Hu W. (2022). Wideband back-cover antenna design using dual characteristic modes with high isolation for 5G MIMO smartphone. IEEE Trans. Antennas Propag..

[19] Tian X., Wang J., Zheng C., Du Z. (2024). Shared-radiator wideband grounded patch antenna pair with bending slot for mobile terminals. IEEE Trans. Antennas Propag..

[20] Tian X., Du Z. (2023). Wideband shared-radiator four-element MIMO antenna module for 5G mobile terminals. IEEE Trans. Antennas Propag..

[21] Chang L., Wang H. (2021). Miniaturized wideband four-antenna module based on dual-mode PIFA for 5G 4 × 4 MIMO applications. IEEE Trans. Antennas Propag..

[22] Cheng S.-H., Chen S.-C., Huang W.-Y. (2025). Low-profile MIMO trapezoidal patch antenna for 5G wideband mobile antenna application. IEEE Antennas Wirel. Propag. Lett..

[23] Hu M., Li Y. (2023). Wideband back cover microstrip antenna with multiple shorting vias for mobile 5G MIMO applications. IEEE Trans. Antennas Propag..

[24] Li K., Zhang Y., Li Y. (2024). Hepta-mode terminal microstrip antenna for mobile Wi-Fi 6/6E and UWB channels 5-11 MIMO applications. IEEE Trans. Antennas Propag..

[25] Lamkaddem A., Merino E., El Yousfi A., Gonzalez-Posadas V., Segovia-Vargas D. Broadband Dual-Polarized Vivaldi Base Station Array Antenna for 5G Smart Networks. Proceedings of the 2023 International Workshop on Antenna Technology (iWAT).

[26] Ansys HFSS (High Frequency Structure Simulator). http://www.ansys.com/products/electronics/ansys-hfss.

[27] Jaglan N., Gupta S.D., Sharawi M.S. (2021). 18 Element Massive MIMO/Diversity 5G Smartphones Antenna Design for Sub-6 GHz LTE Bands 42/43 Applications. IEEE Open J. Antennas Propag..

[28] Khan M.K., Liu S., Khan M.I. (2025). A wideband eight-port MIMO antenna with reduced mutual coupling for future 5G mm-wave applications. Sensors.

[29] (2013). IEEE Recommended Practice for Determining the Peak Spatial-Average Specific Absorption Rate (SAR) in the Human Head from Wireless Communications Devices: Measurement Techniques.

[30] Shoaib S., Bhowmike N., Zahid M., Ali Q., Amin Y., Khattak R. (2026). Highly isolated massive MIMO antennas for future-generation golden band and IoT applications. Arab. J. Sci. Eng..

[31] Ding X.-H., Yang W.-W., Tang H., Guo L., Chen J.-X. (2022). A dual-band shared-aperture antenna for microwave and millimeter-wave applications in 5G wireless communication. IEEE Trans. Antennas Propag..

[32] Tran-Huy D., Tran-Viet-Duc N., Tran H., Hussain N. (2025). A compact MIMO antenna with high gain and dual circular polarization using a T divider for WLAN applications. Sci. Rep..

[33] Khalid M., Iffat Naqvi S., Hussain N., Rahman M., Fawad, Mirjavadi S.S., Khan M.J., Amin Y. (2020). 4-Port MIMO antenna with defected ground structure for 5G millimeter wave applications. Electronics.

[34] Liu M., Wen Y., Wang H., Feng T., Li H. (2026). Broadband Low-Profile Patch Antenna With Efficiency-Optimized Coupling for 5G Mobile Terminals. IEEE Antennas Wirel. Propag. Lett..

[35] Wang X.-Y., Zhu J.-Q., Sun Q., Ban Y.-L. (2026). Optically Transparent U6G MIMO Antenna for Smartphone Applications. IEEE Antennas Wirel. Propag. Lett..

